# Stability of bacteriophages in spray‐dried polymeric formulations: Effect of excipient polyvinylpyrrolidone glass transition temperature and molecular weight

**DOI:** 10.1002/btm2.70096

**Published:** 2026-02-12

**Authors:** Mengyu Li, Yue Cao, Hak‐Kim Chan

**Affiliations:** ^1^ Advanced Drug Delivery Group, Sydney Pharmacy School, Faculty of Medicine and Health The University of Sydney Sydney New South Wales Australia

**Keywords:** bacteriophage (phage) therapy, glass transition temperature (Tg), polymer‐based formulation, polyvinylpyrrolidone (PVP), powder, stability

## Abstract

Effective dry‐state stabilization of bacteriophages is crucial for expanding their therapeutic use. This study builds on previous findings that saccharide‐based formulations maintain phage stability when the glass transition temperature (Tg) exceeds the storage temperature (Ts) by approximately 50°C. We investigated polymer‐based matrices for long‐term stabilization by spray‐drying PEV1 phage with polyvinylpyrrolidone (PVP) of varying molecular weights (K15, K25, K40, K100) and storing for 180 days at temperatures (4, 22, and 40°C) and relative humidity (15%, 33%, 43%, and 53% RH). All formulations achieved minimal titre losses (≤1 log_10_) at 4 and 22°C under 15% RH. Differential scanning calorimetry (DSC) demonstrated Tg increased with PVP molecular weight but decreased substantially with humidity (30–50°C reduction per 20% RH increase). At 33% RH, long‐term stability was achieved with high‐molecular‐weight PVPs (K40, K100), which maintained thermal offsets between Tg and Ts (ΔT ≥ 100°C), while lower‐weight (K15, K25) showed 2–3 log_10_ titre loss. Water activity (*aw*) analysis revealed a critical threshold at *aw* 0.43, above which degradation kinetics increased by approximately a tenfold rate. Arrhenius analysis confirmed that phage degradation rates increased with temperature, consistent with thermally activated destabilization mechanisms. Under higher stress conditions (40°C/≥43% RH), water absorption plasticized the PVP matrix and depressed the Tg while elevated temperature simultaneously accelerated degradation kinetics, resulting in substantial titre losses even when ΔT exceeded 100°C. In conclusion, at mild humidity (*aw* ≤ 0.33) and ambient temperature (≤22°C), high‐molecular‐weight PVP‐based formulations can offer enhanced storage flexibility and reduced cold‐chain dependency for optimal therapeutic viability.

## Graphical abstract

Spray‐dried bacteriophages embedded in polyvinylpyrrolidone (PVP) matrices are stabilised by enforcing a glassy‐state design (Tg – Ts ≥ 100°C), defining a formulation space in which high‐molecular‐weight PVP and controlled water activity enable long‐term phage stability and reduced dependence on the cold chain.

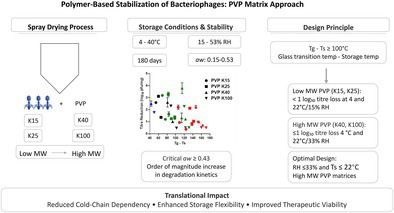

**Translational Impact Statement**

Phage products have already received EMA and FDA approvals for food safety and compassionate use in humans, underscoring their growing antimicrobial relevance. Dry‐state stabilization of phages with reduced cold‐chain dependency is essential for expanding therapeutic applications. This study establishes a novel polymer‐based formulation strategy using high‐molecular‐weight polyvinylpyrrolidone, which preserves phage viability under ambient temperature and moderate humidity. These findings support long‐term storage and distribution of stable phage powders, advancing the development of accessible phage‐based therapies.

## 1 INTRODUCTION

Recent advances in bacteriophage (phage) therapy for antibiotic‐resistant bacterial infections have driven the development of phage powder formulations.^1^, ^2^ Spray‐drying has emerged as a viable approach for producing phage powders, with demonstrated feasibility both in vitro and in vivo.^3^, ^4^ The critical challenge in phage powder development lies in preserving biological activity during processing and storage. The spray‐drying process exposes phages to thermal, osmotic, and shear stresses that can cause structural damage and viability loss.^5^ Protective excipients are essential to maintain phage infectivity in the dry state. While disaccharides such as lactose have shown promise in phage formulations,^6^ alternative polymeric stabilizers like polyvinylpyrrolidone (PVP) offer unique advantages including superior processing characteristics and matrix‐forming properties.^7^, ^8^

The mechanisms of biologic stabilization in solid formulations are generally explained by two established hypotheses: water replacement and vitrification.^9^, ^10^ In the water replacement hypothesis, excipient molecules form hydrogen bonds with proteins to replace water interactions lost during dehydration, maintaining the protein structure.^11^ This mechanism requires the excipient to remain in an amorphous state, as crystallization reduces molecular interactions between excipient and protein, leading to structural damage.^12^, ^13^ The vitrification hypothesis proposes that biologics are immobilized within a rigid, glassy matrix formed by the excipient. The protective capacity of this glassy state is characterized by the glass transition temperature (Tg), above which the matrix transitions from rigid glass to mobile rubber, potentially compromising stability.^9^ Studies on protein formulations have established that maintaining storage temperature at least 50°C below glass transition temperature (Tg) ensures adequate protection through reduced molecular mobility.^14^, ^15^

Environmental factors, particularly humidity, can significantly affect matrix properties through plasticization effects. Increased humidity reduces Tg values, potentially compromising the protective glassy state even when storage temperature remains constant.^16^ This humidity dependence has been demonstrated in protein formulations, where moisture‐induced Tg depression correlates with increased degradation rates.^17^ Despite the success of using glass state in protein stabilization, its systematic application to phage formulations remains largely unexplored. Protein stability in amorphous solids depends on the specific structural and physicochemical features of each protein, which can influence their susceptibility to desiccation‐induced damage.^18^ The larger size and structural complexity of phages may necessitate higher thermal offsets or different excipient properties for adequate protection.

Limited studies have investigated the relationship between Tg and phage stability in powder formulations. Vandenheuvel et al. demonstrated that recrystallization of trehalose‐based phage powders at elevated humidity resulted in up to 3 log_10_ titre reduction, highlighting the importance of maintaining amorphous matrices.^19^ More recently, our previous work on phage powder stabilization established the fundamental relationship between glass transition temperature and phage bioactivity of lactose‐leucine formulations.^20^ This study demonstrated that maintaining storage temperature below Tg was critical for phage stability, with optimal protection achieved when thermal offsets ΔT exceeded 50°C. However, the investigation focused on sugar‐based excipient systems only.

Building upon these foundational findings, the present study extends the glass transition approach to polymer‐based formulations to explore the influence of polymer molecular weight on phage stabilization. Polyvinylpyrrolidone (PVP) offers advantages for phage formulation due to its ability to form stable amorphous matrices across a range of molecular weights. Different PVP molecular weights (K15, K25, K40, K100) exhibit varying Tg values and matrix properties, potentially providing tenable stabilization characteristics.^21^ The hydrogen‐bonding capacity of PVP may contribute to both water replacement and vitrification mechanisms,^22^ though the relative importance of these effects in phage stabilization remains unclear.

The present study investigated the stability of PEV1 phages in spray‐dried formulations containing PVP excipients of varying molecular weights. We systematically evaluated the validity of established ΔT thresholds for phage stability while characterizing molecular weight‐dependent effects on matrix properties and long‐term stability. The research integrated thermodynamic analysis of glass transition behavior with kinetic evaluation of degradation processes to establish design principles for phage powder formulations. Our approach examined whether the ~50°C thermal offset proven effective for phage stabilization in sugar‐based formulations applies to polymeric systems, while identifying critical humidity thresholds and molecular weight effects that govern formulation performance. The findings provided insights into phage‐polymer relationships and established a framework for developing stable phage therapeutics with extended shelf‐life suitable for clinical applications.

## 2 MATERIALS AND METHODS

### 2.1 Materials

Polyvinylpyrrolidone excipients of varying molecular weights (PVP K15, K25, K40, and K100) were purchased from Sigma‐Aldrich (St. Louis, MO). Agar and nutrient broth were supplied by Amyl Media Pty Ltd. The lytic phage PEV1, specific against *Pseudomonas aeruginosa*, was sourced from AmpliPhi Biosciences AU (Australia) with an initial concentration of approximately 10^10^ PFU/mL.

### 2.2 Phage powders

Spray‐dried phage powders were prepared following the methodology described in our previous work.^1^, ^4^, ^5^ Briefly, the liquid feed consisted of 0.5 mL of a phage suspension (approximately 10^10^ PFU/mL) combined with 50 mL of the excipient solution containing either 100% w/w PVP K15, PVP K25, PVP K40, or PVP K100 (pH adjusted to 7.4), yielding a total solid concentration of 25 mg/mL. The term “100% w/w PVP” indicates that PVP was the sole excipient in the formulation, with no additional stabilizing agents. The 25 mg/mL solid content was achieved by dissolving the appropriate weight of PVP in phosphate‐buffered saline (PBS), calculated based on the total mass of solids per volume of solution. The phage titre of the suspension was determined prior to spray drying using a standard plaque assay. The mixture was then spray‐dried using a Büchi Mini Spray Dryer B‐290 (Buchi Labortechnik AG, Flawil, Switzerland) equipped with a standard two‐fluid atomizing nozzle. The spray‐drying parameters were: a liquid feed rate of 1.9 mL/min, an atomizing airflow of 742 L/h, an aspiration rate of 35 m^3^/h, an inlet temperature of 60°C, and outlet temperature maintained between 40 and 41°C. The resulting powders were collected and aliquoted into scintillation vials for storage. To determine the phage titre in the powders, samples were reconstituted in phosphate‐buffered saline (PBS), and phage titres were quantified by plaque assay.

### 2.3 Phage stability

Phage stability was assessed using a standard double‐layer agar method. Briefly, 0.2 mL of an overnight culture of *Pseudomonas aeruginosa* strain PAV237 (~2 × 10^8^ CFU/mL) was combined with 5 mL of molten nutrient broth top agar (0.4% w/v) and poured onto nutrient agar plates (1.5% w/v). After solidification, aliquots (10 μL) of serially diluted phage suspensions were spotted onto the surface of the agar overlay, allowed to air‐dry for approximately 15 min, and incubated at 37°C for 18 h. Each assay was performed independently in triplicate. Phages were classified as stable if the observed titre loss was below 1 log10_10_. Statistical significance of the observed differences was evaluated by two‐way ANOVA, with results considered statistically significant at *p* < 0.05.

### 2.4 Storage conditions

The phage powders were stored following the methodology described in our previous work. Aliquoted phage powders (200 mg per vial) were stored uncapped at controlled temperatures (4, 22, or 40°C) and relative humidity (RH) conditions (15%, 33%, 43%, or 53% RH) for 7 days to allow equilibration of the powders. Following equilibration, the powders continued to be stored under these conditions for up to 180 days, except for conditions at 40°C combined with either 43% or 53% RH, which were excluded from extended studies due to significant titre loss observed within 7 days. To avoid unintended moisture uptake during handling, powders were managed within humidity‐controlled acrylic boxes at either 15% or 33% RH. The chosen storage conditions intentionally deviated from standard ICH guidelines,^23^ reflecting the study's specific aim to explore the mechanistic basis of phage stability by inducing differential changes in Tg across formulations. Specific humidity environments were established using various saturated salt solutions: silica beads provided 15% RH, saturated magnesium chloride solution 33% RH, potassium carbonate 43% RH, and magnesium nitrate 53% RH. These saturated salt solutions were prepared by dissolving excess magnesium chloride, potassium carbonate, or magnesium nitrate in distilled water at 40°C under continuous stirring until saturation. Solutions were subsequently cooled to ambient temperature and sealed in airtight containers for 24 h to equilibrate. Saturated solutions were then placed into vacuum‐sealed containers equipped with humidity sensors for 7 days to confirm stable humidity levels. Once the target RH was consistently achieved, phage powders were prepared and placed into the humidity‐controlled boxes containing the respective saturated solutions.

### 2.5 X‐ray diffraction analysis

The crystallinity of the powder formulations was analyzed using X‐ray powder diffraction (XRPD) with an X'Pert PRO diffractometer (PANalytical, Almelo, the Netherlands) under ambient conditions. Samples were packed into glass capillary tubes (internal diameter: 1.0 mm; WJM‐Glas, Berlin, Germany). The diffraction patterns were obtained using Cu Kα radiation (45 kV, 40 mA), recording scattered intensities over a 2θ range of 3°–50° with an angular increment of 0.028° 2θ per second.

### 2.6 Scanning electron microscopy

The morphology of spray‐dried particles was characterized using scanning electron microscopy (SEM; Zeiss Ultra Plus, Carl Zeiss NTS GmbH, Oberkochen, Germany). Samples were fixed onto aluminum stubs using adhesive carbon tape and subsequently sputter‐coated with gold (approximately 15 nm thickness) utilizing a K550X sputter coater (Quorum Emitech, UK).

### 2.7 Differential scanning calorimeter

Tg of different grades of PVP were determined using modulated differential scanning calorimetry (MDSC) with a DSC 823e instrument (Mettler Toledo, Greifensee, Switzerland). All formulations underwent 7‐day equilibration periods under their respective storage conditions to permit complete Tg adjustment according to environmental temperature and humidity parameters. For each grade, samples weighing 5 ± 1 mg were prepared in sealed aluminum pans. Samples were heated from 30 to 200°C at a heating rate of 2°C/min, employing a modulation amplitude of ±0.5°C and a modulation period of 60 s. Each sample was analyzed independently in duplicate.

### 2.8 Phage degradation kinetics

Phage degradation kinetics were evaluated based on changes in viable phage titre (PFU/mg) over time under various storage conditions. An apparent first‐order kinetic model was used to describe the rate of phage inactivation. The degradation rate constant (k, in day^−1^) was calculated using the equation:

$$\ln\left(\frac{N}{N_{0}}\right)=-\mathrm{kt},$$

where *N* is the phage titre at time *t*, *N*₀ is the initial titre, and *k* is the degradation rate constant (day^−1^).

To assess temperature dependence, *k* values were calculated at each of the storage conditions (4, 22, and 40°C at 15% and 33% RH) over a 30‐day period and fitted to the Arrhenius equation:

$$\ln\left(k\right)=\ln\left(A\right)-\frac{E_{a}}{\mathrm{RT}},$$

where *A* is the pre‐exponential factor, *Eₐ* is the activation energy (J/mol), *R* is the universal gas constant (8.314 J/mol·K), and *T* is absolute temperature (K).

The influence of moisture was further assessed by plotting *k* as a function of water activity (*aw*), estimated from the equilibrium relative humidity (RH) using the relationship *aw* ≈ RH/100 (i.e., RH values of 15%, 33%, 43%, and 53% correspond to *aw* ≈ 0.15, 0.33, 0.43, and 0.53, respectively).

## 3 RESULTS

### 3.1 Physicochemical characterization of spray‐dried formulations

**FIGURE 1:**
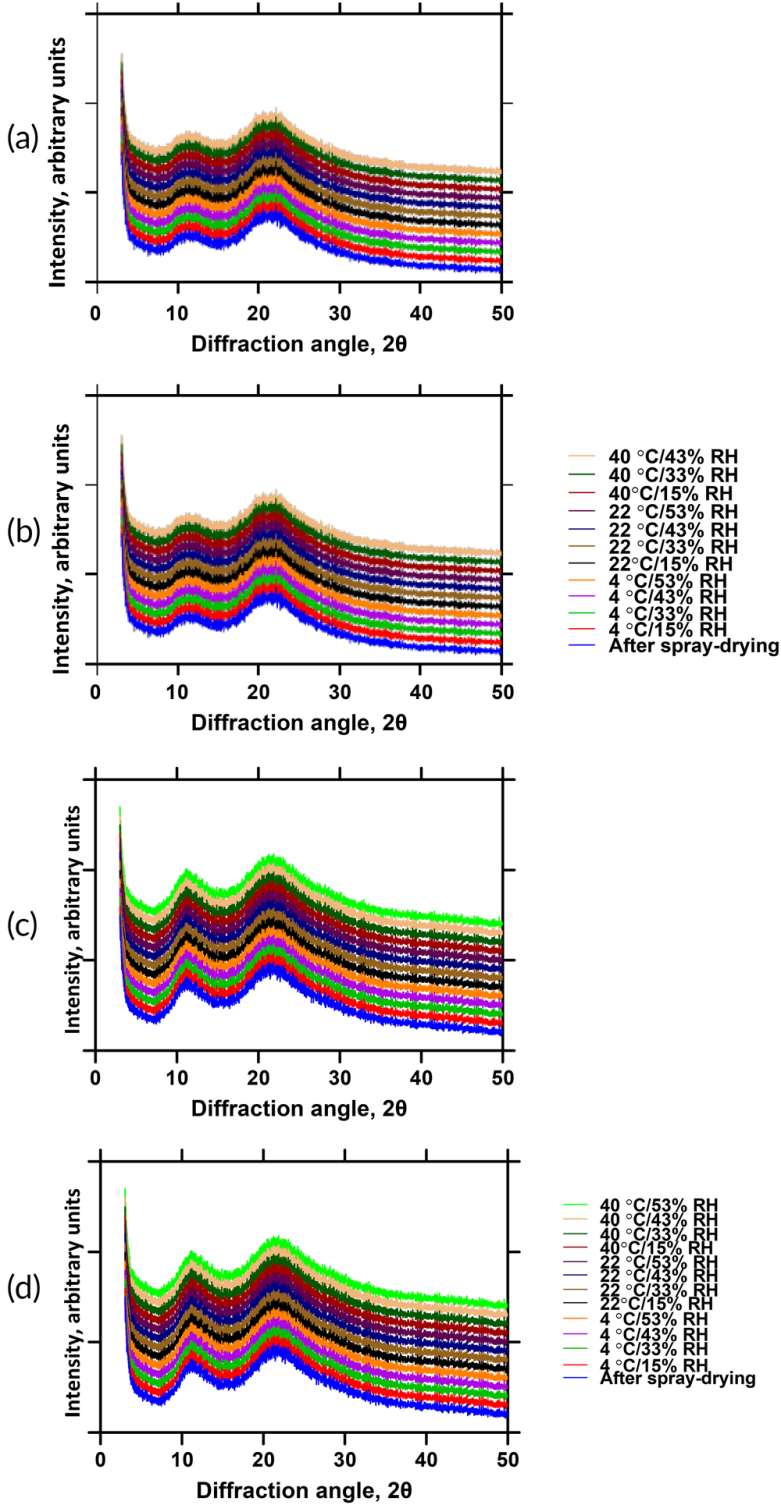
X‐ray diffraction (XRD) patterns of spray‐dried phage formulations with PVP K15 (a), PVP K25 (b), PVP K40 (c), and PVP K100 (d) after 7‐day storage at various temperatures and relative humidities.

The solid‐state and morphological properties of the spray‐dried phage formulations were characterized. X‐ray powder diffraction analysis of all formulations containing PVP of varying molecular weights (K15 to K100) confirmed the absence of crystalline peaks after a 7‐day equilibration period across all tested temperatures (4, 22, and 40°C) and relative humidity conditions (15%, 33%, 43%, and 53% RH) (Figure 1).

**FIGURE 2:**
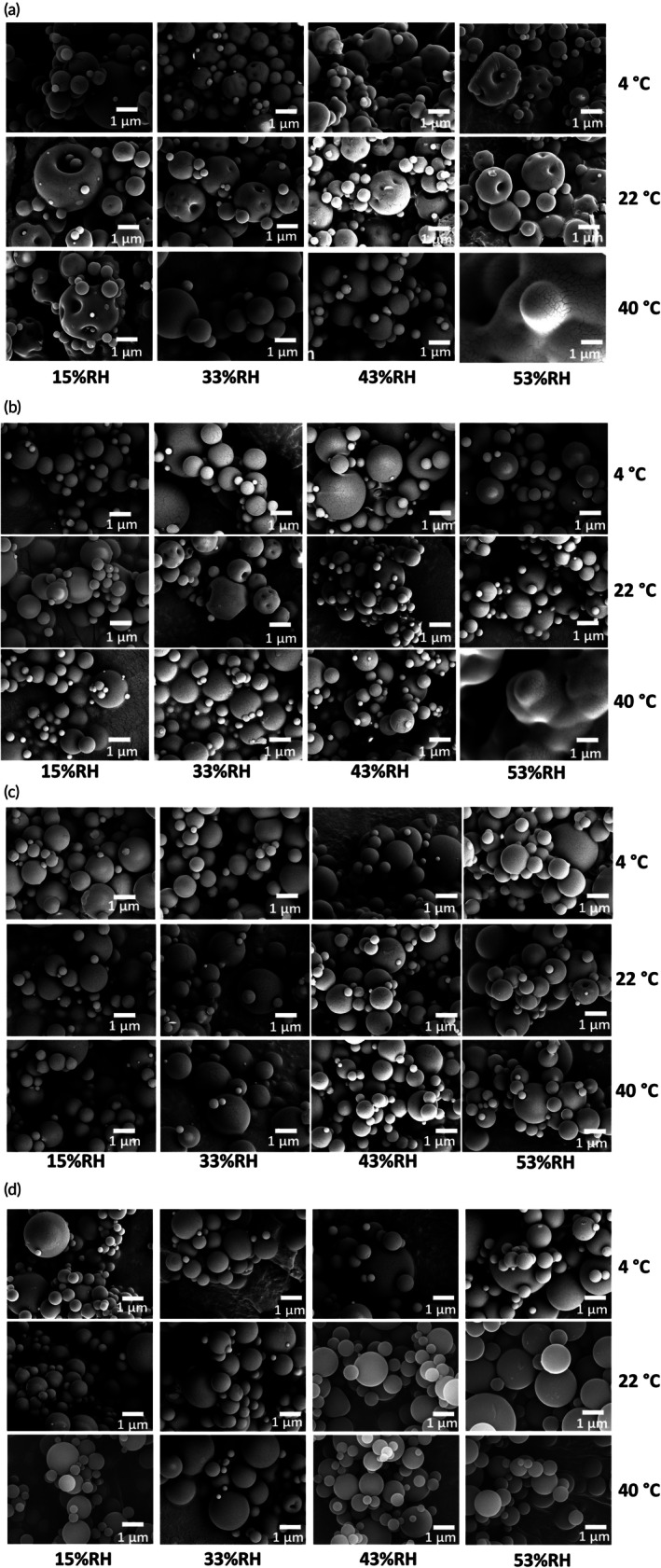
Scanning electron microscopy (SEM) images of spray‐dried phage formulations with PVP K15 (a), PVP K25 (b), PVP K40 (c), and PVP K100 (d) after 7‐day storage at various temperatures and relative humidities.

SEM revealed that the particle morphology was dependent on the molecular weight of the PVP excipient and the storage conditions (Figure 2). PVP K15 formulations exhibited dimpled and irregular particle surfaces at 4 and 22°C with 15–33% RH, and extensive particle fusion at 40°C/53% RH. PVP K25 formulations showed generally smooth and spherical morphologies at lower temperatures and RH, but also displayed signs of fusion at 40°C/53% RH. PVP K40 and K100 formulations maintained well‐defined, discrete spherical particles across all tested conditions. In particular, PVP K100 particles stored at 40°C/53% RH appeared notably smooth and uniform, with fewer visible surface irregularities or fusion compared to lower molecular weight formulations under the same conditions.

The initial Tg of the formulations under dry conditions (15% RH) increased proportionally with the molecular weight of the PVP, ranging from 124°C for the PVP K15 formulation to 171°C for the PVP K100 formulation (Table 1). Exposure to elevated humidity systematically depressed the Tg values. An increase in RH from 15% to 33% lowered the Tg by approximately 40°C, with a further increase to 43% RH causing an additional 25–30°C reduction.

**TABLE 1:** Tg of phage powders after 7 days under various storage conditions.

Excipient	Storage RH (%)	Tg at 4°C (°C)	Tg at 22°C (°C)	Tg at 40°C (°C)
PVP K15	15	128 ± 0.2	131 ± 0.6	147 ± 0.8
	33	88 ± 0.5	98 ± 0.2	109 ± 0.2
	43	58 ± 0.2	N/A	N/A
	53	N/A	N/A	N/A
PVP K25	15	142 ± 0.2	164 ± 0.2	173 ± 0.7
	33	102 ± 0.1	112 ± 0.3	123 ± 0.2
	43	70 ± 0.2	82 ± 0.2	N/A
	53	N/A	N/A	N/A
PVP K40	15	167 ± 0.2	176 ± 2.7	187 ± 0.2
	33	127 ± 0.7	136 ± 0.2	155 ± 0.8
	43	97 ± 0.2	103 ± 0.5	N/A
	53	47 ± 0.2	N/A	N/A
PVP K100	15	175 ± 0.9	182 ± 0.8	192 ± 0.8
	33	135 ± 0.2	145 ± 0.2	160 ± 1.1
	43	106 ± 0.2	111 ± 0.2	N/A
	53	54 ± 0.3	N/A	N/A

Abbreviation: N/A, not applicable.

### 3.2 Phage viability following processing and storage

#### 3.2.1 Initial processing and short‐term stability (7 days)

**FIGURE 3:**
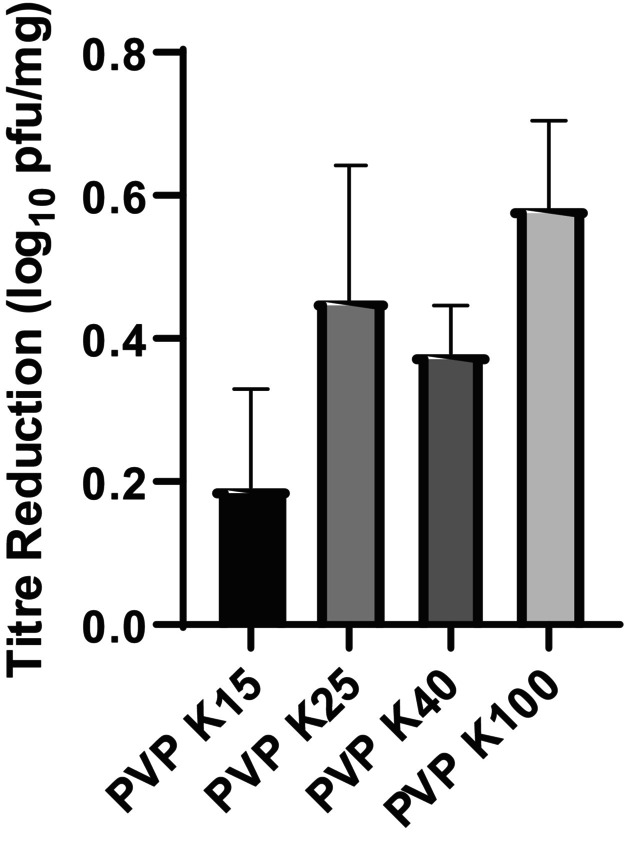
Title loss of PEV1 after spray‐drying process (*n* = 3).

The spray‐drying process itself resulted in minimal loss of phage viability, with all formulations retaining titre losses below 1 log_10_. The specific losses were 0.20 (K15), 0.45 (K25), 0.38 (K40), and 0.58 (K100) (Figure 3).

**FIGURE 4:**
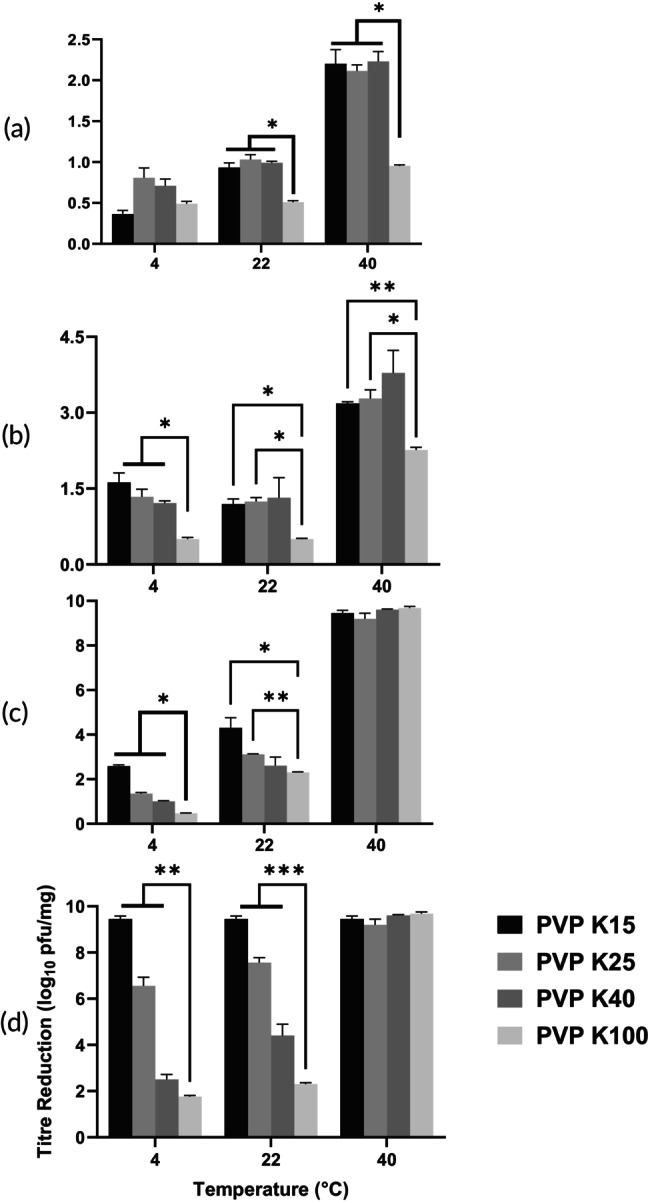
Titre loss of PEV1 powders with PVP K15, K25, K40, and K100 after 7‐day storage at 4, 22, or 40°C and 15% RH (a), 33% RH (b), 43% RH (c), or 53% RH (d), presented as mean ± SD (*n* = 3). Statistical significance was assessed using two‐way ANOVA. *p < 0.05; **p < 0.01; ***p < 0.001; n.s., not significant.

After 7 days of storage, phage viability was dependent on a combination of PVP molecular weight, temperature, and RH (Figure 4). At 15% RH, titre losses remained below 1 log_10_ for all formulations at 4 and 22°C. However, at 40°C, the PVP K100 formulation exhibited a 0.95 log_10_ loss, compared to losses of 2.1–2.23 log_10_ for the lower molecular weight grades. At elevated humidity (53% RH and 4°C), a clear trend emerged where titre loss was inversely proportional to PVP molecular weight, ranging from 1.76 log_10_ for PVP K100 to 9.45 log_10_ for PVP K15. At the most strenuous conditions (40°C and ≥43% RH), all formulations experienced titre losses greater than 9 log_10_.

#### 3.2.2 Long‐term stability (180 days)

**FIGURE 5:**
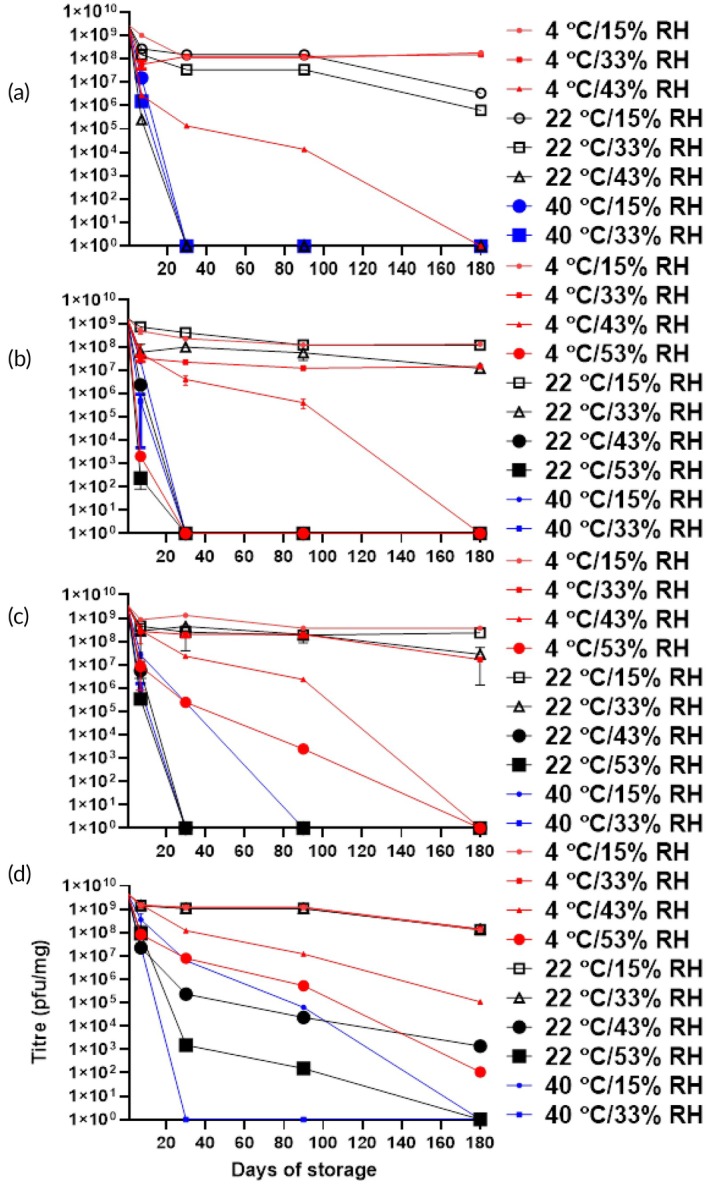
Title of PEV1 powders with PVP K15 (a), PVP K25 (b), PVP K40 (c), and PVP K100 (d) after storage at various temperatures and relative humidities, presented as mean ± SD (*n* = 3).

Extended storage for 180 days showed viability trends consistent with the short‐term data (Figure 5). All formulations maintained high phage titres (~10^8^ PFU/mg) for 180 days when stored at 4°C under both 15% and 33% relative humidity. At 22°C and 33% RH, the PVP K100 formulation also preserved high titres (~10^8^ PFU/mg) over 180 days, while the PVP K40 formulation maintained titres ranging from ~10^8^ to 10^9^ PFU/mg under the same conditions. In contrast, the viability of lower molecular weight formulations declined under more challenging conditions. For instance, the PVP K25 formulation titre fell to <1 PFU/mg by 180 days at 4°C/43% RH, while the PVP K15 formulation titre fell to <1 PFU/mg under the same conditions by Day 90.

### 3.3 Correlation of stability with physicochemical parameters

**FIGURE 6:**
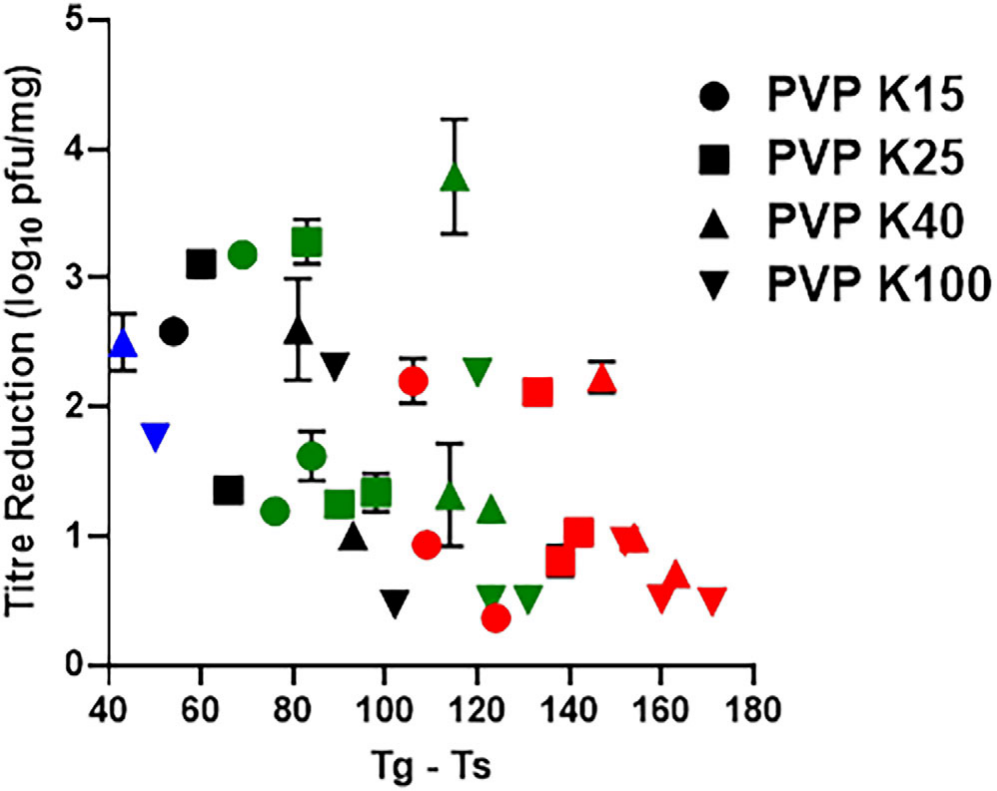
Scatterplot of glass transition temperature (Tg) minus storage temperature (Ts) versus log_10_ titre loss of PEV1, with 15% RH (red), 33% RH (green), 43% RH (black), and 53% RH (blue).

The relationship between the physical state of the matrix and phage stability was investigated by correlating phage titre loss with the thermal offset (ΔT = Tg − Ts). As presented in Figure 6 and detailed in Table 2, a clear inverse relationship was observed between the calculated ΔT value and the measured loss in phage titre after a 7‐day equilibration period.

**TABLE 2:** Difference between Tg and Ts of phage powders after 7 days under various storage conditions.

Excipient	Storage RH (%)	Tg − Ts at 4°C (°C)	Tg − Ts at 22°C (°C)	Tg − Ts at 40°C (°C)
PVP K15	15	124	109	107
	33	84	76	69
	43	54	N/A	N/A
	53	N/A	N/A	N/A
PVP K25	15	138	142	133
	33	98	90	83
	43	66	60	N/A
	53	N/A	N/A	N/A
PVP K40	15	163	154	147
	33	123	114	115
	43	93	81	N/A
	53	43	N/A	N/A
PVP K100	15	171	160	152
	33	131	123	120
	43	102	89	N/A
	53	50	N/A	N/A

Abbreviation: N/A, not applicable.

At 15% RH, where ΔT values were high (102–171°C), the corresponding titre losses were less than 2.3 log_10_ across all formulations. In contrast, at 33% RH, the ΔT values were lower, ranging from 69 to 131°C. Under these conditions, measured titre losses increased significantly to a range of 0.50–3.79 log_10_. It was also noted that at comparable ΔT values, the K100 formulation provided superior protection (0.50 log_10_ loss at ΔT = 131°C) compared to lower molecular weight grades (3.18–3.79 log_10_ loss).

**FIGURE 7:**
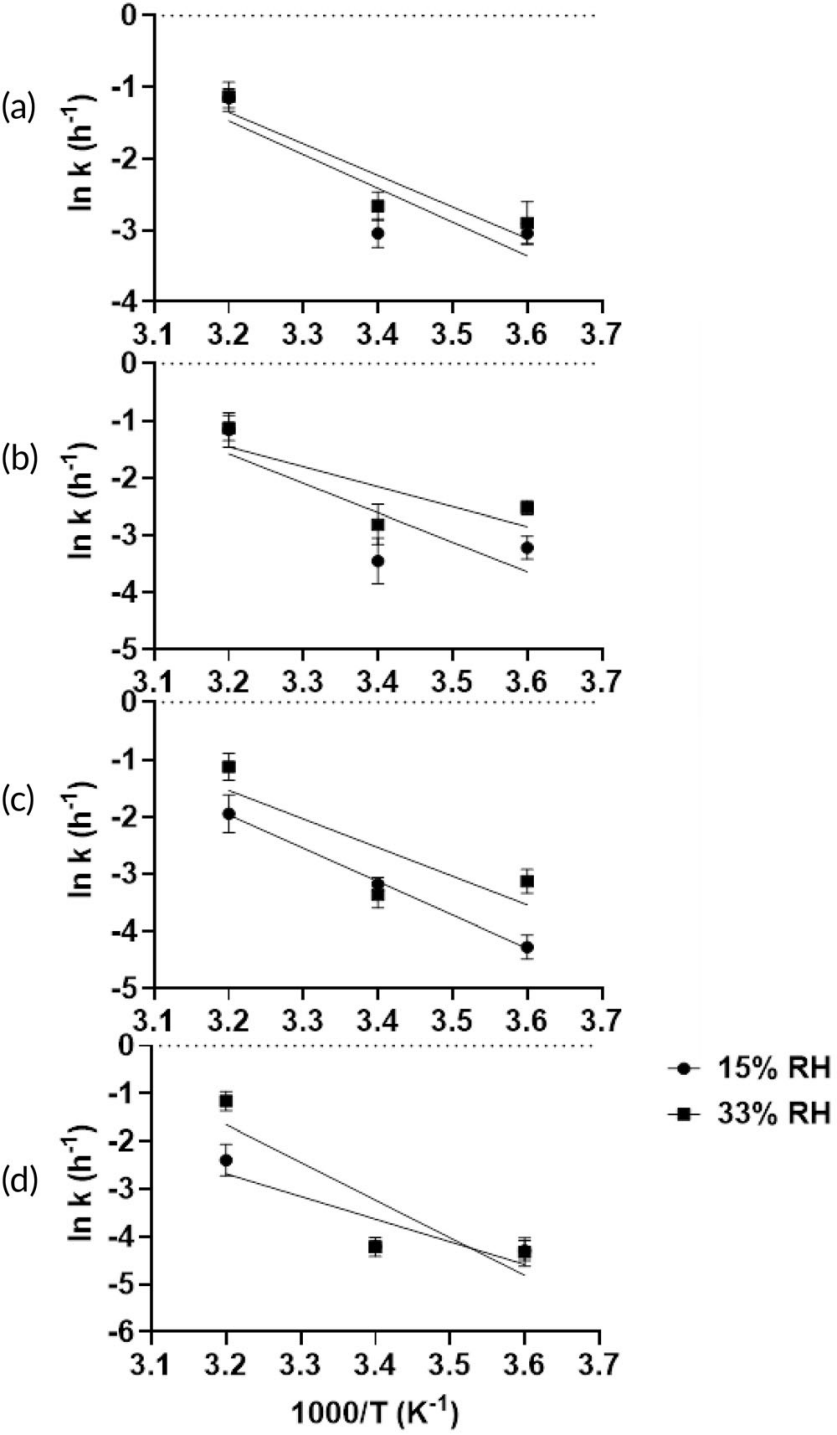
The Arrhenius plots for the degradation rate of spray‐dried PEV1 over 30 days stored at 15% and 33% RH for each formulation: PVP K15 (a), PVP K25 (b), PVP K40 (c), and PVP K100 (d).

**FIGURE 8:**
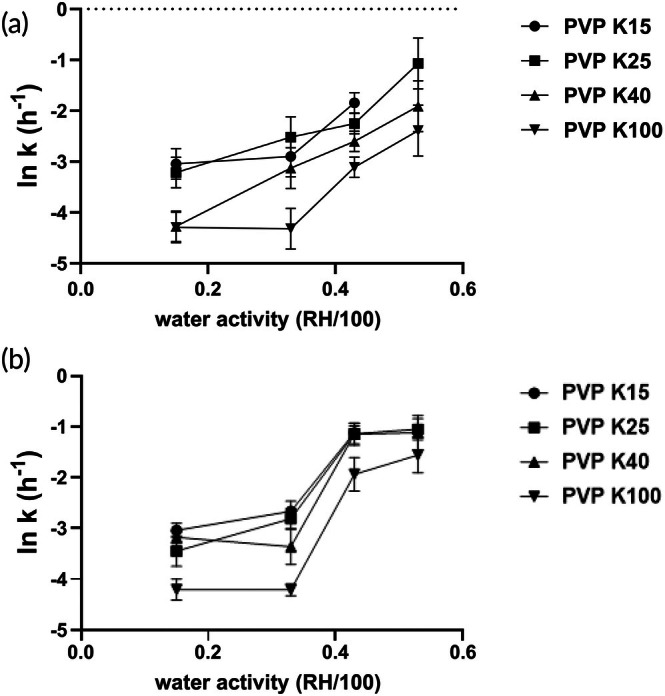
The effect of water activity on the degradation rate of spray‐dried phage formulations at 4°C (a) and 22°C (b).

To further characterize this temperature‐dependent degradation, the degradation rate constants (*k*) were determined at 15% and 33% RH. The degradation was found to follow first‐order kinetics, as indicated by the linear relationship in Arrhenius plots of ln(*k*) versus inverse temperature (*R* ^2^ = 0.60–0.99), shown in Figure 7. The effect of *aw* on the degradation rate of spray‐dried PEV1 powders was evaluated at 4 and 22°C (Figure 8). At both temperatures, the degradation rate constant (*k*) increased as *aw* rose from 0.15 to 0.43. Between *aw* 0.15 and 0.33, ln(*k*) values showed minimal variation for all formulations. A pronounced increase in ln(*k*) was observed when *aw* reached 0.43, particularly at 22°C, where ln(*k*) values approached −1.1 for PVP K15–K40 and −1.9 for PVP K100.

## 4 DISCUSSION

In this study, we aimed to investigate PVP‐based polymeric matrices for stabilization of spray‐dried phage formulations, as a suitable alternative to conventional saccharide systems across various storage conditions. Building upon previous findings from Chang et al., which demonstrated that having a ΔT of approximately 50°C using saccharide‐based excipients maintained phage stability for up to 250 days under controlled conditions,^20^ we sought to evaluate whether polymer‐based systems could offer enhanced storage stability, particularly under elevated humidity conditions that typically compromise saccharide formulations. Our systematic evaluation focused on glass transition temperature, humidity‐dependent degradation kinetics, and the effects of polymer molecular weight (K15, K25, K40, K100) across storage temperatures ranging from 4 to 40°C and RH values of 15%–53% over a 180‐day period. Our analysis revealed that all PVP formulations achieved equivalent stability at 4 and 22°C under low humidity (15% RH). However, under elevated humidity (33% RH), only PVP K100 consistently achieved the higher thermal offsets required for phage preservation, whereas K40 did not perform better than lower‐MW variants.

PVP has proven stabilizing properties for biologics in pharmaceutical formulations.^24^ PVP having both hydrophilic and hydrophobic components may counteract this by adsorbing at the interface, reducing surface tension, and preventing phage adsorption, thus protecting them within the droplet core.^25^ Additionally, PVP in the dry state could stabilize phages through vitrification, forming a glassy matrix that limits molecular mobility and prevents capsid unfolding.^26^ Its ability to form hydrogen bonds with phage proteins could potentially compensate for water loss during drying, preserving structural integrity.^27^ In our study, all PVP variants effectively preserved PEV1 phage viability immediately post spray‐drying, with the titre losses all below 1 log_10_, ranging from 0.20 (K15) to 0.58 (K100) (Figure 3). The differences in titre losses between formulations, spanning less than 0.5 log_10_, are within the typical variability of plaque assays, indicating that these minor variations are not statistically significant and do not reflect a meaningful trend among the PVP variants. These low losses demonstrate PVP's suitability as a stabilizer during the spray‐drying process.

The thermal offset ΔT emerged as a decisive factor in maintaining phage stability in dry formulations containing saccharides as an excipient. The identification of ΔT ≥50°C as the critical threshold for stabilizing phage in these formulations represents the minimum thermal offset required to preserve the rigid glassy state that restricts degradative molecular processes.^28^ Molecular relaxation processes become increasingly constrained as temperature decreases below Tg, which improves the likelihood for biological macromolecules to maintain their structural integrity.^29^ These findings align with established principles for the stabilization of protein formulation, where maintaining storage temperature at least 50°C below Tg ensures adequate protection through reduced molecular mobility.^30^ Short‐term (7‐day) studies showed that PVP formulations with a ΔT well over 100°C effectively preserved phage bioactivity, with minimal titre losses (<1 log_10_) at low temperature and humidity (4°C/15% RH) (Figure 4). However, ΔT alone was not a complete predictor of stability. At elevated temperatures (40°C), only the K100 formulation maintained low titre loss, while other PVP variants with a ΔT > 50°C showed significant degradation (*p* < 0.05), suggesting other microenvironmental factors are influential. Long‐term stability (180 days), which is vital for commercial viability, mirrored short‐term results at lower temperatures. Formulations with a substantial ΔT stored at 4 and 22°C retained high phage titres (Figure 5). Conversely, long‐term stability at elevated temperatures was poor, even with a high ΔT. For instance, the K100 formulation at 40°C/15% RH (ΔT = 112°C) lost nearly all titre by 180 days. Studies on phage thermal stability indicate that some phages lose infectivity at temperatures above 40°C, even in stabilized forms, due to structural damage.^31^ Analysis of Arrhenius degradation kinetics across the 4–40°C temperature range demonstrated linear relationships for all PVP formulations over the initial 30 days, with goodness‐of‐fit coefficients (*R* ^2^ = 0.60–0.99) confirming the applicability of apparent first‐order kinetics (Figure 7).

Additionally, this study observed that higher‐molecular‐weight PVPs produced more uniform particle morphologies as revealed by SEM (Figure 2). The distinct particle morphology observed for PVP K100, particularly the smooth and spherical shapes retained under high humidity and temperature conditions, reflects the influence of polymer molecular weight on droplet shell formation during spray drying. The final morphology of a spray‐dried particle is governed by the mechanical strength of the crust that forms on the droplet surface as solvent evaporates. The ability of this crust to withstand internal pressure gradients during drying determines whether the particle retains its spherical shape or collapses into a wrinkled form.^32^ In formulations containing low‐molecular‐weight PVPs, the shorter polymer chains form a brittle crust that is prone to buckling, resulting in the irregular and deformed particle surfaces observed for K15 and K25. In contrast, the long‐chain, high‐molecular‐weight PVP (K100) forms a more viscoelastic and mechanically resilient crust that resists collapse during drying, enabling the preservation of a smooth and spherical particle morphology.

The impact of humidity on glass transition temperature is an important factor governing phage stability in the PVP formulations. DSC analysis revealed substantial Tg decreases with increasing humidity across all molecular weight PVP samples. Under the dry condition (15% RH) at 4°C, Tg values ranged from 124°C for PVP K15 to 171°C for PVP K100 (Table 1). At 33% RH, Tg decreased by approximately 40°C in all PVP samples, while further humidity increased to 43% RH produced an additional 25–30°C depression (Table 1). Each 20% increase in relative humidity resulted in a 30–50°C reduction in Tg across this PVP molecular weight series, demonstrating the pronounced plasticization effect of moisture on polymer matrices. High humidity posed challenges for long‐term phage stability, even with a ΔT > 100°C. At 4°C and 53% RH, PVP K100 exhibited a titre reduction to 110 PFU/mg (~7 log_10_ loss) by 180 days, with a ΔT of ~80°C (Table 2 and Figure 5). Similarly, at 22°C and 43% RH (estimated ΔT ≈ 70°C), titres dropped to 1400 PFU/mg (~6 log_10_ loss). The *aw* dependence of degradation kinetics provides mechanistic insight into humidity‐mediated destabilization of phage formulations.^33^ Kinetic analysis (Figure 8) revealed that increasing *aw* from 0.15 to 0.33 produced only modest changes in the degradation constant (ln *k* ≤ −2.7 for all PVP formulations), indicating that the glassy matrix maintains reasonable protective capacity under low to mild humidity conditions. However, a critical threshold emerges at *aw* 0.43, where the degradation kinetics increased by a tenfold increase in the degradation rate constant, as reflected by the ln *k* values of approximately −1.1 for K15 to K40 and −1.9 for K100 (Figure 8). At *aw* 0.53, the degradation rate remained elevated, yielding ln *k* values of −1.1 for K25–K40 and −1.6 for K100 (Figure 8). The increased *aw* likely plasticizes the polymeric matrix, enhancing molecular mobility of PVP and facilitating degradation of phage, as commonly observed in studies where water acts as a plasticizer in amorphous solids of small molecule drugs and biologics.^16^, ^34^

Polymeric molecular weight impacts Tg and, consequently, phage stability in formulations. Our results demonstrated a clear correlation between increasing molecular weight of PVP and higher Tg values (Table 1), due to enhanced polymer chain entanglement and reduced molecular mobility.^28^, ^35^ The elevated Tg associated with higher molecular weight PVPs translated directly into improved phage stability. At 53% RH and 4°C, titre losses increased significantly with decreasing molecular weight after 7 days of storage: 1.76 log_10_ (K100), 2.50 log_10_ (K40), 6.55 log_10_ (K25), and 9.45 log_10_ (K15) (*p* < 0.01) (Figure 4). At 40°C and 15% RH, PVP K100, with a ΔT of 112°C, showed a loss of 0.95 log_10_, compared with >2 log_10_ in K15, K25, and K40, indicating that higher molecular weight PVPs enhance stability even at elevated temperatures (Table 2 and Figure 4). PVP K100 maintained titres of approximately 10^9^ PFU/mg at 4 and 22°C under 15% and 33% RH after 180 days (Figure 5). At the more humid conditions of 53% RH at 4°C, a detectable titre (110 PFU/mg) after 180 days was still retained in the K100 sample, but not in the lower molecular weight PVP (K15, K25, K40). We proposed that in the higher molecular weight K100, extended polymer chains provide more extensive hydrogen bonding opportunities with phage surface proteins, creating stabilizing networks of intermolecular interactions that facilitate maintenance of protein tertiary structure.^27^ Higher molecular weight PVP matrices exhibit increased entanglement density, which generates more tortuous diffusion pathways.^36^ This structural complexity acts as a barrier to the ingress of mobile species such as water molecules, thereby offering enhanced protection against moisture‐induced degradation and preserving phage integrity.^36^ Our study demonstrates that the stability of spray‐dried phages is fundamentally linked to the physical properties of the polymer matrix. We found that high‐molecular weight (MW) PVP, which promotes a high glass transition temperature (Tg) and extensive polymer chain entanglement, creates a robust vitrified matrix. This structure immobilizes the virions and impedes moisture ingress, thereby kinetically limiting degradation pathways and enhancing long‐term phage viability. Beyond Tg alone, these stabilizing effects arise from the interplay of matrix rigidity, tortuosity, and intermolecular interactions. For instance, increased chain entanglement in high‐MW PVPs leads to more mechanically resilient glassy structures that are less prone to plasticization, while simultaneously restricting the diffusion of water and degradative species. These multiple mechanisms act synergistically to reduce the vulnerability of entrapped phages under storage stress. Based on this established mechanism, we anticipate that other polymers may offer similar protective benefits. Polymers capable of forming amorphous, high‐Tg matrices with long‐chain entanglement—such as high‐MW polyvinyl alcohol (PVA) or polyethylene oxide (PEO)—may replicate these stabilization effects. Exploring such alternatives could expand the design space for phage dry powder formulations, particularly when balancing stability requirements with regulatory, processing, or delivery considerations.

Our previous study using a lactose‐leucine matrix^20^ showed ~3 log_10_ titre loss under 33% RH, due to moisture‐induced Tg depression (~30°C), which lowered ΔT to below 50°C. This was attributed to increased matrix mobility due to moisture‐induced plasticization. PVP formulations with ΔT values well above 100°C exhibited minimal titre loss, and this superior stability—relative to the lactose system—reflects fundamental differences between polymer‐based and saccharide‐based glasses. PVP forms physically entangled polymer networks that inherently restrict chain mobility and confer high intrinsic Tg.^16^, ^37^ These structural features allow PVP matrices to maintain a rigid glassy state and low molecular mobility, even under moderate humidity, thereby preserving the stability of embedded biologics. In contrast, saccharide‐based glasses—such as those formed by trehalose or lactose—are composed of small, highly soluble molecules that tend to form more homogeneous, amorphous solids without chain entanglement. Although these matrices can also vitrify under dry conditions, they are more susceptible to moisture uptake due to their hygroscopic nature. The absorbed water acts as a plasticizer, significantly lowering Tg and increasing molecular mobility. As a result, saccharide systems often require a safety margin in ΔT.^38^ In our current study, all PVP‐based formulations maintained high phage titres at 4°C and 33% RH, consistent with large ΔT values. However, only the high‐MW formulations remained effective at 22°C and 33% RH, suggesting a key role of polymer molecular weight in maintaining stability under moderate humidity. We propose that tightly bound water in high‐MW PVP may contribute to virion stabilization without plasticizing the matrix. The dense, entangled structure of high‐MW polymers not only resists moisture‐induced Tg suppression but may also retain small amounts of structurally bound water, which can help preserve capsid hydration without compromising glassy integrity. This dual function is consistent with previous reports highlighting the protective role of residual water in protein formulations.^39^ In contrast, low‐MW PVPs, which exhibit greater free volume and chain mobility, are more susceptible to water‐induced plasticization. In such systems, absorbed water behaves more as a plasticizer than a stabilizer, leading to reduced protective capacity. These findings reinforce the importance of ΔT as a predictor of stability, while highlighting that for polymer‐based systems like PVP, both Tg and molecular structure are critical determinants of long‐term preservation under ambient or humid conditions. While the molecular mechanisms underlying these effects were not directly investigated in this study, they align with the observed trends. Future work will apply nanoscale analytical techniques (e.g., AFM‐IR and SNOM) and solid‐state NMR,^40^, ^41^ to further elucidate the interactions among the polymer, water, and phage within the glassy matrix.

This study provides valuable insights into phage stabilization using PVP‐based formulations, but several limitations remain. The findings are specific to PEV1, and it is unclear whether they generalize to other phages with different structural and stability profiles. Longer‐term studies beyond 180 days are also needed to assess clinical shelf‐life feasibility. Unexpected titre losses in certain conditions (e.g., PVP K40 at 22°C/33% RH despite ΔT = 115°C) suggest that factors beyond ΔT, such as phage–polymer interactions or localized degradation pathways, may influence stability. Moreover, similar Tg values across PVP variants under humid conditions limit differentiation, highlighting the need to explore excipients with distinct thermal behaviors. Hygroscopicity was not directly measured in this study; techniques such as DVS or TGA should be applied to better understand moisture uptake. While this study focused on Tg measurements after equilibration to specific storage conditions, time‐course analysis of Tg evolution during long‐term storage may provide further insight into moisture uptake dynamics and should be explored in future studies. Finally, phage degradation under extreme conditions (40°C/43–53% RH), despite ΔT > 100°C, suggests that thermal effects beyond bulk vitrification contribute to instability. Future research should address these limitations by testing diverse phages, conducting extended stability studies, and investigating underlying mechanisms to optimize phage powder formulations.

## 5 CONCLUSIONS

This study presents the first systematic investigation of PVP‐based polymer matrices for phage stabilization, establishing novel formulation principles distinct from saccharide‐based systems. All PVP formulations preserved phage viability within 1 log_10_ loss over 180 days when stored at 4 and 22°C under 15% RH. At higher humidity (33% RH), long‐term stability was successfully achieved at ambient temperatures (≤22°C) using high‐molecular‐weight PVPs (K40, K100), which provided the necessary thermal offsets (ΔT ≥ 100°C) to maintain a protective glassy state. These findings introduce humidity‐tolerant design parameters for phage formulation in PVP that allow long‐term phage stability storage and reduce cold‐chain dependency.

## AUTHOR CONTRIBUTIONS

Mengyu Li conceived the project and wrote or revised the manuscript. Hak‐Kim Chan supervised the research. Yue Cao was responsible for sample collection, preparation, and revised the manuscript. Mengyu Li conducted data analysis.

## CONFLICT OF INTEREST STATEMENT

The authors declare no conflicts of interest.

## Data Availability

The data that support the findings of this study are available from the corresponding author upon reasonable request.
